# Venous Thromboembolic Complications in Pediatric Gastrointestinal Diseases: Inflammatory Bowel Disease and Intestinal Failure

**DOI:** 10.3389/fped.2022.885876

**Published:** 2022-04-28

**Authors:** Renz C. W. Klomberg, Lotte E. Vlug, Barbara A. E. de Koning, Lissy de Ridder

**Affiliations:** Division of Gastroenterology, Department of Pediatrics, Erasmus MC Sophia Children's Hospital, University Medical Center Rotterdam, Rotterdam, Netherlands

**Keywords:** thrombosis, Crohn's disease, ulcerative colitis, short bowel syndrome, thromboprophylaxis, anticoagulation, children, gastroenterology

## Abstract

In children with gastrointestinal disorders such as inflammatory bowel disease (IBD) and intestinal failure (IF), the risk of venous thromboembolism (VTE) is increased. VTE may lead to pulmonary embolism, sepsis and central line infection, stroke and post-thrombotic syndrome. The purpose of this review is to summarize current knowledge and recent advances around VTE management in pediatric gastroenterology with a focus on IBD and IF. The VTE incidence in children with IBD is reported to be around 4–30 per 10,000 patient-years, with higher incidences for hospitalized children. While in general, IF is less common than IBD, the VTE incidence in children with IF is around 750 per 10,000 patient-years. The most common risk factors for development of VTE involve deviations leading to Virchow's triad (endothelial damage, stasis, and hypercoagulability) and include active inflammation, particularly with colonic involvement, presence of a central venous catheter, underlying thrombophilia, reduced mobility, surgery, and hospitalization. Classes of anticoagulants used for treatment of VTE are low molecular weight heparins and vitamin K antagonists. However, the use of direct oral anticoagulants for treatment or prevention of VTE has not been studied in this pediatric population yet. Pediatric gastroenterologists apply different VTE prevention and treatment strategies due to lack of literature and lack of consensus. We discuss the role of primary and secondary prophylactic use of anticoagulants, and provide tools and recommendations for screening, prevention and management for the specific pediatric populations.

## Introduction

Venous thromboembolism (VTE) involves deep venous thrombosis, pulmonary embolism, cerebral sinovenous thrombosis (CSVT), central venous catheter (CVC) related VTE, and abdominal vein thrombosis, such as portal, or mesenteric vein thrombosis (1). According to Virchow's triad (dating from 1856), VTE results from endothelial damage, stasis, and hypercoagulability (2). In general, VTE may lead to post-thrombotic syndrome and chronic thromboembolic pulmonary hypertension, associated with substantial morbidity and high healthcare expenses (3).

Although VTE is rare in childhood, pediatricians, and more specific pediatric gastroenterologists, may encounter children with VTE, either with gastrointestinal symptoms or consequences of VTE, or with VTE as complication of gastrointestinal diseases. Abdominal vein thrombosis, which can be secondary to intra-abdominal infections, pancreatitis, surgery, and sickle cell disease, is often asymptomatic, but may eventually manifest with symptoms of acute abdomen, or with gastrointestinal complications reflecting portal hypertension, including liver dysfunction, splenomegaly, or upper gastrointestinal bleeding (4). On the other hand, gastrointestinal diseases that may be complicated by development of VTE are cystic fibrosis, celiac disease, inflammatory bowel disease (IBD) and intestinal failure (IF) (5, 6). Within this spectrum, children with IBD and IF are most at risk of developing VTE.

The estimated prevalence of IBD is 58.9–66.3 per 100,000 children in Western Europe (7). The worldwide incidence is increasing, especially in younger children (7–9). Recently, emerging data has demonstrated that VTE is associated with IBD in children, but the precise mechanism is not yet fully understood. VTE in children with IBD is associated with longer hospital stay, increased need for intensive care unit admission, higher costs, and increased in-hospital mortality (10).

In children with IF, the gut insufficiently absorbs nutrients and fluids needed for growth and maintaining homeostasis (11). Therefore, these children depend on long-term parenteral nutrition (PN) given through a CVC. Prevalence of long-term PN varies between 9.6 and 14.1 per million children (12, 13). Children with IF may develop VTE, in IF mostly referred to as central venous thrombosis (CVT), because it is related to the presence of a CVC (14). Recurrent VTE is a major barrier for delivering PN and can result in loss of vascular access. Loss of vascular access is one of the criteria for consideration of small bowel transplantation (15).

Given the risk and consequences of VTE in children with IBD and IF, pediatric gastroenterologists need practical tools for diagnostics, prevention and treatment of VTE in these populations, while considering disease-specific risk factors and underlying mechanisms. Therefore, this review aims to describe incidence, mechanisms and risk factors of VTE, and to outline screening, prevention and treatment strategies for VTE in pediatric gastroenterology with a focus on IBD and IF. We offer insight into controversies, current research gaps and new developments in the field.

## Incidence

In a recent meta-analysis, VTE incidence in the general pediatric population was found to be 0.27 per 10,000 person-years (PY) (16).

### Inflammatory Bowel Disease

In recent years, several studies have evaluated the VTE risk in children with IBD. A Canadian population-based study reported a 5-year incidence of 31.2 per 10,000 PY for VTE, compared to 0.78 per 10,000 PY in children without IBD (17). The incidence was highest in the 1st year after diagnosis (81.2 per 10,000 PY). Another Danish population-based study found an incidence rate of 8.9 per 10,000 PY for VTE in children with IBD, with a hazard ratio of 6.6 compared to age-matched controls. The prospective international Safety Registry, covering 24,802 children with IBD, reported an incidence of 3.09 per 10,000 PY, 14-fold higher than the incidence rate in the general pediatric population (16). Altogether, these studies clearly show an increased VTE risk in children with IBD, though with varying incidence rates, which is likely the result of a different methodology and background population of the studies. Interestingly, CSVT has been described as a relatively common VTE type in children with IBD. According to two studies, including a systematic review, CSVTs made up to almost 50% of VTE cases, with a reported incidence rate of 1.86 per 10,000 PY in the Safety Registry (16, 18). Another single-center retrospective study found that in 4/154 (2.6%) newly-diagnosed children with IBD a CSVT occurred within 5 years. Although this was not supported by data from other pediatric studies (17, 19), physicians should be vigilant about CSVT occurrence in children with IBD, especially considering the significant morbidity and mortality.

### Intestinal Failure

Although pediatric IF is much less prevalent than pediatric IBD (PIBD), VTE itself is much more common in children with IF. Reported incidences of VTE vary among studies, depending on the population studied, the follow-up duration period and the time since diagnosis of IF. In a recent meta-analysis (from 2019) including 1,277 children with IF, the authors reported thrombosis to be present in 25.7%, resulting in an event rate of 7.5% per year and an incidence of 750 per 10,000 PY (14). When looking at individual studies published after publication of the meta-analysis, ranging prevalences have been reported. Asouzo et al. reported one or more thrombi in 30 patients from a cohort of 65 patients with IF (46%) with a median age of 6.83 years (20), while Schmidt et al. reported thrombosis in 95 out of 388 patients with IF (24%) during a median follow-up time of 398 days (21). Secondary (i.e., recurrent) VTE occurred in 44% of the patients of the latter study. LaRusso et al. retrospectively included 37 patients with IF, of which 8 developed venous thrombosis (22%) (22).

## Pathophysiology

### Pathophysiology in Inflammatory Bowel Disease

**Figure 1:**
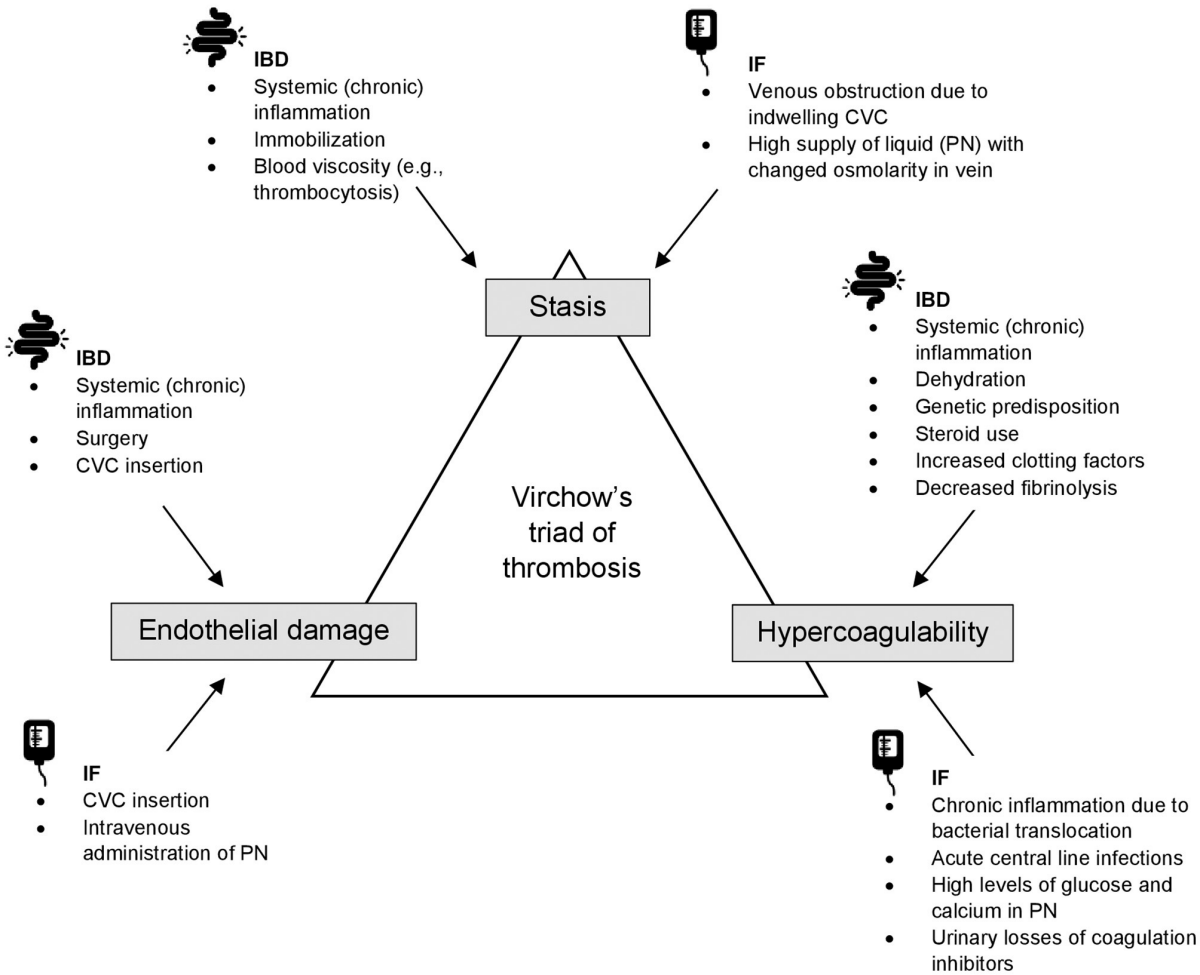
An overview of Virchow's triad of thrombosis in IBD and IF. CVC, central venous catheter; IBD, inflammatory bowel disease; IF, intestinal failure; PN, parenteral nutrition.

In PIBD, the increased VTE risk results from a combination of increased prevalence of general VTE-related risk factors and prothrombotic conditions in this patient population, such as immobilization or dehydration, and the systemic inflammation leading to the development of Virchow's triad (Figure 1). Prothrombotic abnormalities observed in IBD include initiation and activation of the coagulation system (e.g., increased coagulants such as fibrinogen, factor V, VIII, IX, Von Willebrand factor or thrombin-antithrombin complexes), reduced activity of natural anticoagulant mechanisms (e.g., decreased antithrombin III, decreased protein C and S, and decreased tissue factor pathway inhibitor), abnormality of fibrinolysis, platelet abnormalities (thrombocytosis, increased activation and aggregation), and reactivity and dysfunction of the endothelium (23–26). Prothrombogenic cytokines that are implicated to be the driver of thrombus formation in patients with IBD are IL-1b, TNF-a, and IL6 (23).

### Risk Factors in Inflammatory Bowel Disease

Several risk factors may contribute to the higher VTE risk in children with IBD (Table 1). Generally, VTE is more common in adolescents than in younger children (27). So far, there is no evidence confirming this is also the case in PIBD.

**Table 1:** Risk factors for developing venous thromboembolic events in children with inflammatory bowel disease and/or intestinal failure.

**General demographic/patient risk factors**	**Disease-specific risk factors**	
	**Inflammatory bowel disease**	**Intestinal failure**
• Thrombophilia (acquired/hereditary)	• Active inflammatory disease	• Underlying disease: NEC/gastroschisis
• Previous VTE	• (Extensive) colonic involvement	• Low parental education
• Malignancy	• Corticosteroid use	• Number of CVC replacements
• Immobility	• Recent surgery	• Number of CVC related blood stream infections
• Infection		• Increased CVC lumen diameter
• Oral anticonception use		• Long indwelling time of CVC
• Dehydration		• PN macronutrient and micronutrient composition

*CVC, central venous catheter; NEC, necrotizing enterocolitis; PN, parenteral nutrition; VTE, venous thromboembolism*.

#### Hospitalization

By far one of the most precipitating factors is hospitalization. One study reported an incidence of 117.9 per 10,000 PY in hospitalized children with IBD (28). The Safety Registry study reported a relative risk of 559 for hospitalization (16). However, VTE development is not limited to hospitalized patients. According to a recently published study, VTE was the indication for admission in 11/23 (48%) cases (29), and in the Safety Registry study, 9/21 (43%) cases occurred in non-hospitalized patients (16). Therefore, awareness of VTE occurrence and prevention should not be limited to admitted patients.

#### Active Disease

Active inflammation is another important factor contributing to increased VTE risk in patients with IBD. This was already proven by several large adult studies (30, 31) and has recently been demonstrated in a pediatric study, in which all but one patient had clinical or biochemical evidence of active disease (16).

#### Surgery

Another independent risk factor is surgery. McKie et al. demonstrated that children with IBD undergoing a major surgical procedure had a 1.98 greater odds of developing a VTE, compared to children with IBD without major surgery (32). Bence et al. performed a retrospective review of children who underwent colorectal resection for IBD and found that 4.3% of patients developed postoperative VTE after a median of 14 days postoperatively (33). The increased risk of VTE after surgery might even extend after hospital-discharge, as has been demonstrated in adults with ulcerative colitis (UC) after urgent surgery (34).

#### Ulcerative Colitis

Pediatric UC patients are likely to be more prone to thromboembolic events than Crohn's disease (CD) patients. A meta-analysis in adults showed similar risks in patients with UC and CD (RR 2.57 vs. 2.12) (35), while several studies in children found a lower incidence rate in CD patients (17, 19). The risk difference between adult and pediatric UC patients could be explained by the fact that children more often present with pancolitis, which is more extensive disease and has been associated with VTE in adults with IBD (36, 37). In the Safety Registry, all six CD patients had colonic involvement and all 14 UC patients had pancolitis (16). These findings suggest that extensive colonic involvement confers a higher risk of VTE, though the association with endoscopic disease severity has not been evaluated yet.

#### IBD-Related Treatment

There is little evidence regarding the relationship between IBD therapy and VTE risk in children with IBD. In the previously mentioned Safety Registry, 9/20 patients received corticosteroids, and 4/20 received anti-TNF prior to the VTE event (16). Another nested case-control study in hospitalized patients with IBD reported that steroid use was an independent risk factor of VTE (OR 12.7) (29). To the best of our knowledge, no other studies assessed the association between VTE risk in children with IBD and therapy use. Generally, corticosteroids are considered thrombogenic. Steroids contribute to the hypercoagulable state by increasing levels of factor VIII/von Willebrand factor complex and by inducing a hypofibrinolytic state by increasing plasminogen activator inhibitor 1 (38). In adults, corticosteroid use is associated with a higher risk of VTE (OR 2.2) (39). However, this could be secondary to active disease rather than corticosteroid use alone. There is conflicting data regarding the risk of VTE in adults with IBD receiving biological therapy, such as TNF-alfa inhibitors. Sarlos et al. suggested a 5-fold decreased VTE risk of TNF-alfa inhibitors compared to steroids (39). Another nationwide observational study showed no difference in VTE risk between patients with IBD treated with TNF-alfa inhibitors compared with non-biologic immunomodulating agents (e.g., thiopurines, methotrexate), though in a subgroup of patients <45 years, TNF-alfa inhibitors were associated with VTE risk reduction in CD patients (40).

#### Other IBD-Related Risk Factors

Several unspecific risk factors that are common in patients with IBD may increase the risk of VTE. These include dehydration due to increased gastrointestinal losses and impaired nutritional status, presence of CVCs, total PN and reduced mobilization due to active disease or fatigue.

### Pathophysiology in Intestinal Failure

In pediatric IF, the increased VTE risk likely results from vessel wall injury due to CVC insertion and intravenous administration of PN. Also, a hypercoagulable state may be present due to chronic inflammation, acute central line infections, and high levels of glucose and calcium in PN (41). Proteinuria, which is common in children with IF on long-term PN (42), is accompanied by urinary losses of coagulation inhibitors such as antithrombin and protein S and C, also leading to a hypercoagulable state (43). In Figure 1, mechanisms in IF leading to the development of Virchow's triad are presented.

### Risk Factors in Intestinal Failure

VTE risk factors in IF (Table 1) are mostly related to the CVC. VTE may be due to catheter insertion and PN, multiple surgical procedures to replace catheters and repeated episodes of CVC related blood stream infections (44–46). LaRusso et al. found no significant difference in thrombosis rates between different types of catheters used: in tunneled CVC use, incidence was 0.15/1,000 catheter days; and in peripherally inserted central catheter (PICC) use, incidence was 0.55/1,000 catheter days (22). In another study, not only CVC related risk factors were found, but VTE was also found to be associated with parental education (20). Possibly, in parents with lower education, there is a lack of understanding of line care and delay in recognition of line complications and infection. Another risk factor is underlying disease, in which necrotizing enterocolitis and gastroschisis were predictors of VTE (20). This may be because these diseases involve inflammation of the gut. Inflammation increases fibrinogen concentration, induces tissue factor expression on the cell surface of leucocytes, and increases platelet production through inflammatory mediators such as interleukin-6; all involved in the coagulation cascade (47).

## Management

Table 2 includes special considerations for identification of risk factors, VTE prevention, VTE recognition, and VTE management in children with IBD and IF.

**Table 2:** Special considerations for VTE prevention and management in children with IBD and IF.

**General**
• Timely identification and adequate treatment of concomitant infections.
• Provide adequate hydration and nutritional status.
• Limit use of CVCs, and administer oral/enteral nutrition when possible.
• Promote (early) mobilization, especially post-surgery and during active disease; consider compression stockings for patients with prolonged immobilization.
**Inflammatory bowel disease**
• Screening for hereditary and acquired thrombophilia in children with IBD with a family history of VTE and patients with a previous VTE during inactive disease.
• Consider early diagnosis of pulmonary embolism with CT angiography in patients with signs or symptoms of pulmonary embolism (e.g., shortness of breath, fainting, or chest pain while breathing).
• Consider early diagnosis of CSVT with CT of the brain in patients with signs or symptoms of CSVT (e.g., severe headache, fainting, altered consciousness, altered vision, or seizures).
• Consider thromboprophylaxis for hospitalized patients with active disease, irrespective of age.
**Intestinal failure**
• Consider thromboprophylaxis in all children with long-term PN delivered through a CVC.
• Consider oral/enteral supplementation of vitamin K antagonists instead of subcutaneous injection of LMWH in children aged ≥1 year.
• Consider routine annual ultrasound screening of head, neck, and arm veins for VTE in children without thromboprophylaxis.

*CSVT, cerebral sinus venous thrombosis; CT, computed tomography; CVC, central venous catheter; IBD, inflammatory bowel disease; IF, intestinal failure; LMWH, low-molecular-weight heparin; PN, parenteral nutrition; VTE, venous thromboembolism*.

### Treatment

Treatment of VTE in children with IBD and IF traditionally consists of heparin (unfractionated heparin or low-molecular-weight heparin (LMWH)), followed by LMWH or vitamin K antagonists (VKAs) (48). Direct oral anticoagulants (DOACs), such as apixaban, rivaroxaban and edoxaban (direct factor Xa inhibitors), and dabigatran (direct thrombin inhibitor), offer advantages over these traditional agents, such as oral route of administration, in general no need for laboratory monitoring of anticoagulant activity, and no dose adjustment. The first completed pediatric studies show that DOACs could be a safe and effective alternative for treatment of VTE in children (49). However, it remains unknown if these results can be extrapolated to specific patient groups at risk of worse outcome (e.g., bleeding), such as children with IBD or IF (50). Altered pharmacokinetics or drug interactions in these patients might require dose adjustments and better monitoring by measuring trough or peak plasma concentrations. Absorption of DOACs occurs in different locations, with apixaban being mainly absorbed in the distal small intestine and proximal colon, and edoxaban, rivaroxaban and dabigatran absorbed in the proximal gastrointestinal tract (e.g., stomach and proximal small intestine) (51). Therefore, these latter agents might be an option in stable children with (active) IBD. DOACs seem less useful in children with IF due to absorption problems in the proximal gastrointestinal tract. Despite absorption problems, VKAs are also administered orally in some children with IF, who tolerate a little oral nutrition. Monitoring of INR values ensures that therapeutic VKA levels can be achieved. If children with IF or IBD receive a DOAC, laboratory monitoring is obligatory to guarantee therapeutic anticoagulation levels, depending on the site of absorption and location of the gastro-intestinal disease.

The role of DOACs as thromboprophylaxis in children is yet to be established. When providing thromboprophylaxis with DOACs for children with IBD or IF, the same considerations apply as for treatment of VTE.

### Prevention and Screening

#### Inflammatory Bowel Disease

Genetic risk seems to be associated with development of VTE in patients with IBD (52). The contribution of hereditary or acquired thrombophilia to VTE development in patients with IBD is likely to be limited (16, 32, 53), and routine screening should thus be reserved for unusual cases. To decide which patients might benefit from thromboprophylaxis, personalized risk-stratification based on general and disease-specific VTE risk factors risk factors is needed. However, until recently, there has been a lot of reluctance in prescribing thromboprophylaxis. In a survey among 162 pediatric gastroenterologists, 92% agreed children with IBD are at increased risk for VTE, but only 1/3 of them ever provided prophylaxis for their patients. Eighty-one percent reported this was because of a paucity of data (54). Because of limited evidence, current ESPGHAN guidelines only recommend thromboprophylaxis with low molecular weight heparin in the setting of adolescents with an acute severe colitis (ASC) with additional risk factors (55). Another issue of prescribing thromboprophylaxis is safety concerns, especially the presumed bleeding risk in patients with active disease (54, 56, 57). However, in a retrospective study in children hospitalized for ASC that received enoxaparin (LMWH) as thromboprophylaxis, no differences in hemoglobin levels or need for blood transfusions were observed. (58) Further support for the safety of thromboprophylaxis comes from adult studies with IBD and from a meta-analysis on thromboprophylaxis in children in general (59).

Fortunately, prevention of VTE in PIBD, based on personalized risk-stratification, has recently received more attention. According to a RAND appropriateness panel, prophylactic anticoagulation was deemed appropriate in all children with IBD admitted with ASC during the COVID-19 pandemic (60). A limitation was that the clinical scenarios did not take into consideration specific patient factors and thus VTE risk factors. Therefore, and based on cumulative evidence of VTE risk, another RAND appropriateness panel was convened this year, comprising pediatric gastroenterologists and experts in pediatric surgery and hematology, that rated the appropriateness of thromboprophylaxis in other settings, while taking into consideration VTE risk factors such as age and sex (61). These initiatives highlight the expanding interest of PIBD experts and current advances in the field of VTE prevention. Despite the increased awareness and vigilance of VTE risk in children with IBD, thromboprophylaxis has not yet been routinely used in this high-risk population. This should be done based on personalized risk-stratification, weighing both VTE risk factors and bleeding risk.

#### Intestinal Failure

In 2018, Neelis et al. explored organization and clinical practice of IF teams in an international survey (62). According to the ESPGHAN/ESPEN guidelines on PN in infants, children and adolescents (from 2005, applicable at that time), VKAs or LMWH may be given prophylactically to patients on long-term PN at risk of thromboembolism or with previous thromboembolism (63). According to the survey, different VTE prevention and treatment strategies were applied across Europe: 46% of 59 IF teams prescribed prophylactic anticoagulation. Types of anticoagulation used were LMWH (standard in 14%, sometimes in 19% of the teams), heparin lock (standard in 12%, sometimes in 17% of the teams), and VKAs (standard in 2%, sometimes in 14% of the teams). The main reason for not prescribing prophylactic anticoagulation was lack of evidence. Prophylactic anticoagulation was used significantly more frequently in the teams with >10 patients compared with teams with ≤ 10 patients on home PN (59 vs. 28%, respectively) (62).

Already in 2003, the use of prophylactic warfarin was examined. No new VTE and no bleeding complications were found in this study including eight patients (64). In a study comparing LMWH and VKA prophylaxis with no prophylaxis in 32 patients, less occlusions, less infections, and no bleeding complications were found in the group receiving prophylaxis (65). Recently, Nagelkerke et al. assessed VTE and bleeding incidence in a cohort of 55 children with IF on home PN and prophylactic anticoagulation (LMWH or VKA) (66). Cumulative thrombosis-free survival was 96% after 2 years, and 78% after 5 years. Eight cases of VTE occurred on LMWHs (0.14/1,000 anticoagulation days) and 2 on VKAs (0.17/1,000 anticoagulation days). Cumulative bleeding-free survival was 81% after 2 years, and 73% after 5 years. The incidences of clinically relevant and major bleeding were 0.1 and 0.03 per 1,000 catheter days, respectively. These are relatively low rates of VTE and slightly elevated rates of bleeding, compared with studies in which no prophylaxis was used (65, 67).

It remains unclear what the best anticoagulant regimen is for preventing VTE. In the current ESPGHAN/ESPEN guidelines on venous access in pediatric PN (from 2018), the authors state that there is insufficient evidence to recommend prophylactic anticoagulants (68). We need studies comparing prophylaxis with no prophylaxis by means of randomized controlled trials or by comparing thrombosis and bleeding outcomes between centers with different regimens. In the ESPGHAN/ESPEN guidelines, it is recommended to use the antiseptic agent taurolidine during long-term CVC use to prevent line infections (68). This recommendation is mostly based on evidence in adult studies, but also in children the effectiveness of taurolidine has been shown (69, 70). Since recurrent CVC related blood stream infections are associated with higher risk of CVT development, taurolidine might also prevent CVT indirectly.

There are no guidelines for children with IF when it comes to secondary prophylaxis to prevent recurrent thrombosis. However, in the new NASPGHAN recommendations (from 2021), the authors state that children with incomplete resolution of thrombus should be maintained on prophylactic anticoagulation with LMWH (71). Schmidt et al. retrospectively compared two different regimens for secondary anticoagulation prophylaxis with LMWH in two pediatric IF centers (21). In the short-term regimen, children with CVT received therapeutic dosing until thrombus resolution or up to 3 months without prophylactic dosing thereafter, and in the long-term regimen, therapeutic dosing was given up to 3 months and prophylactic dosing continued thereafter until line removal. The authors recommend long-term secondary anticoagulation prophylaxis in children with CVT that require PN for prolonged periods of time, since secondary thrombosis occurred in eight of 13 (62%) patients in the short-term group and in nine of 26 (35%) in the long-term protocol group (*P* = 0.019) after a median time of 144.5 and 689 days, respectively (*P* = 0.01).

Routine screening for CVT in children on long-term PN is subject of debate. Nagelkerke et al. recently recommended routine, annual radiologic screening for CVT development in these children, since 40% of the CVT they found with ultrasound screening were asymptomatic (66). However, in a study concerning critically ill children with asymptomatic CVT, no radiographic extensions of thrombi during follow-up were seen and acute or long-term complications were rare despite absence of treatment (72). However, in these critically ill children, the CVC was removed because PN was no longer needed, which is not possible for children with IF. Routine radiographic screening could be considered in patients without thromboprophylaxis.

## Conclusion

In this review, we have shown that both children with IBD and IF are at increased risk of VTE compared to the general pediatric population. Risk factors are both catheter-related and disease-specific, but also more generic underlying mechanisms such as thrombophilia, hospitalization and inflammation play a key role in the development of VTE. Once VTE is diagnosed, close collaboration with pediatric hematologists is needed for adequate treatment and subsequent follow-up of VTE. However, as prevention is key, thromboprophylaxis for high-risk patients might be of even more importance. Due to lack of evidence, there is no clear guidance on prevention of VTE in both IBD and IF. Future studies are therefore required to evaluate the safety and efficacy of thromboprophylaxis, including DOACs.

## Author Contributions

RK and LV reviewed the literature and wrote and edited the manuscript. BK and LR supervised the study and revised the manuscript. All authors agreed with the final version of the manuscript.

## Conflict of Interest

The authors declare that the research was conducted in the absence of any commercial or financial relationships that could be construed as a potential conflict of interest.

## Publisher's Note

All claims expressed in this article are solely those of the authors and do not necessarily represent those of their affiliated organizations, or those of the publisher, the editors and the reviewers. Any product that may be evaluated in this article, or claim that may be made by its manufacturer, is not guaranteed or endorsed by the publisher.
